# Detailed prediction of protein sub-nuclear localization

**DOI:** 10.1186/s12859-019-2790-9

**Published:** 2019-04-23

**Authors:** Maria Littmann, Tatyana Goldberg, Sebastian Seitz, Mikael Bodén, Burkhard Rost

**Affiliations:** 1 Department of Informatics, Bioinformatics & Computational Biology - i12, TUM (Technical University of Munich), Boltzmannstr. 3, 85748 Garching/Munich, Germany; 2 School of Chemistry and Molecular Biosciences, UQ (University of Queensland), Cooper Rd, Brisbane City, QLD 4072 Australia; 3 Institute for Advanced Study (TUM-IAS), Lichtenbergstr 2a, 85748 Garching/Munich, Germany; 4 TUM School of Life Sciences Weihenstephan (WZW), Alte Akademie 8, Freising, Germany; 5 Department of Biochemistry and Molecular Biophysics & New York Consortium on Membrane Protein Structure (NYCOMPS), Columbia University, 701 West, 168th Street, New York, NY 10032 USA

**Keywords:** Sub-nuclear localization, Traveler proteins, Prediction, Support vector machines (SVM), Profile kernel, GO enrichment, Evolutionary information, Predict protein function

## Abstract

**Background:**

Sub-nuclear structures or locations are associated with various nuclear processes. Proteins localized in these substructures are important to understand the interior nuclear mechanisms. Despite advances in high-throughput methods, experimental protein annotations remain limited. Predictions of cellular compartments have become very accurate, largely at the expense of leaving out substructures inside the nucleus making a fine-grained analysis impossible.

**Results:**

Here, we present a new method (*LocNuclei*) that predicts nuclear substructures from sequence alone. *LocNuclei* used a string-based Profile Kernel with Support Vector Machines (SVMs). It distinguishes sub-nuclear localization in 13 distinct substructures and distinguishes between nuclear proteins confined to the nucleus and those that are also native to other compartments (traveler proteins). High performance was achieved by implicitly leveraging a large biological knowledge-base in creating predictions by homology-based inference through BLAST. Using this approach, the performance reached AUC = 0.70–0.74 and Q13 = 59–65%. Travelling proteins (nucleus and other) were identified at Q2 = 70–74%. A Gene Ontology (GO) analysis of the enrichment of biological processes revealed that the predicted sub-nuclear compartments matched the expected functionality. Analysis of protein-protein interactions (PPI) show that formation of compartments and functionality of proteins in these compartments highly rely on interactions between proteins. This suggested that the *LocNuclei* predictions carry important information about function. The source code and data sets are available through GitHub: https://github.com/Rostlab/LocNuclei.

**Conclusions:**

LocNuclei predicts subnuclear compartments and traveler proteins accurately. These predictions carry important information about functionality and PPIs.

**Electronic supplementary material:**

The online version of this article (10.1186/s12859-019-2790-9) contains supplementary material, which is available to authorized users.

## Background

The nucleus was the first sub-cellular organelle to be discovered as early as in the seventeenth century [1]. It is enclosed by a membrane and only found in eukaryotic cells (Greek “eu” εν: true, “karyon” καρυον: kernel, i.e. cells with a core, Latin: nucleus). The nucleus contains most of the genetic material, organized in chromosomes, and is the site for DNA replication and transcription. Nuclear proteins are synthesized mostly on the ribosomes in the cytoplasm and have to be transported back into the nucleus for proper function. Import into and export out of the nucleus differ in several ways from the transport to other sub-cellular compartments. For instance, all proteins have to pass through a large structure in the nuclear envelope known as the nuclear pore complex (NPC) [2, 3]. Nuclear proteins can be transported in their fully folded conformation [3]. Transport is often regulated through binding to specific proteins, called *karyopherins*. Karyopherins bind by recognizing nuclear localization signals (NLS for import into the nucleus) or nuclear export signals (NES; for export from the nucleus) in the amino acid sequence of their cargo proteins [4]. Relying only on these NLS and NES fails to identify nuclear proteins because many known signals are too unspecific in sequence (match in many non-nuclear proteins) and for most known nuclear proteins such signals remain unknown [5–7].

The nucleus is a compartment separated by two membranes that contains several distinct sub-structures, each associated with distinct sets of function. These nuclear sub-structures are not enclosed by membranes and are very dynamic. Nuclear sub-structures can be in continuous flux; some are exclusively formed during particular cell stages through interaction with DNA, RNA and proteins [8, 9]. These dynamic rearrangements complicate experimental annotations. Translocation within the nucleus has been linked to NLS- and NES-like signals [10, 11]. However, this process is not well understood [8].

## Results

### High performance: Q13 = 62% and Q2 = 72%

**Fig. 1:**
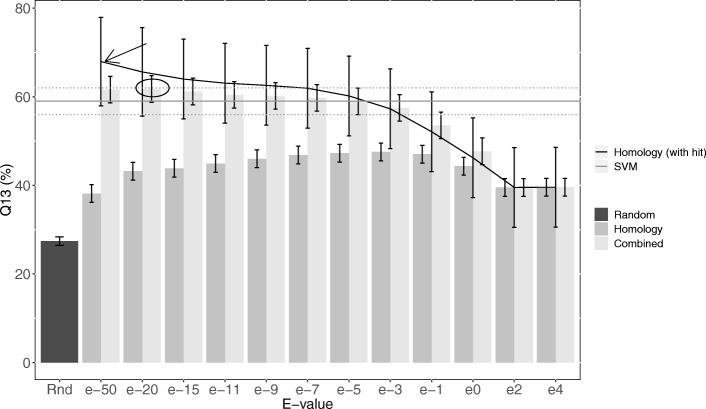
Effect of homology threshold to predict 13 sub-structures. The accuracy Q_13_ for classifying proteins into 13 sub-nuclear compartments using the homology-based inference with PSI-BLAST (based on 3522 experimentally annotated proteins) varied with the E-value thresholds (darker gray bars on the left). For proteins for which a protein with experimentally known nuclear sub-structure annotation was more sequence similar than the threshold, performance depended on the threshold (black line). The highest accuracy Q_13_ = 68% was reached at E-value ≤10^− 50^(black arrow). However, if forcing predictions for all proteins, Q_13_ dropped to 38% compared to random (27%). The performance of machine learning-based profile kernel SVMs on the same set was Q_13_ = 59% (gray horizontal line). The lighter gray bars mark the combination of homology inference and machine learning. The optimal threshold for the combination was E-value ≤10^− 20^. One standard error marked on each bar and on the black line and through the dotted lines for ML

LocNuclei describes two separate prediction methods: (1) predict one of 13 nuclear sub-structures and (2) distinguish proteins functional only in the nucleus vs.traveler proteins,i.e.those functional in the nucleus and other compartments. Each of those two methods combines two different algorithms: (i) homology-based inference and (ii) machine learning-based prediction (through profile kernel SVMs). For the prediction task of 13 sub-nuclear compartments, the homology-based inference for proteins for which experimentally annotated homologs were available was most accurate with Q_13_ = 68% at *E*-value ≤10^− 50^ (Fig. 1 black arrow). However, if only using homology-based inference, a random decision had to be made when no homolog of known localization was available at a given threshold. Thus,the Q_13_ dropped to 38% (Fig. 1: left bar at *E*-value 10^− 50^). This was still statistically significantly above random (Fig. 1: standard error bars substantially above random performance of 27% shown at the leftmost bar). On the same test set, the de novo-based inference employing a battery of 13 SVM classifiers achieved an almost three-fold higher level of Q_13_ = 59% (Fig. 1: 2nd bar from the left). This result encouraged the application of a simple protocol: use homology-based inference when available, else use the machine learning method. The accuracy of homology-based inference decreased for less stringent *E*-value thresholds (Fig. 1: line decreases toward right). We chose the PSI-BLAST *E*-value of 10^− 20^ as the decision threshold between homology-based inference and machine learning based de novo prediction because the simple combination of homology-based and de novo was highest (the performance was determined using cross-validation/cross-training, i.e. NOT the testing set). The combined method, LocNuclei, outperformed both its components (Fig. 1: circle above bar for SVM and homology), reaching an overall accuracy of Q_13_ = 62 ± 3% (Fig. 1: circle).

In terms of relative contributions of HB vs. ML for our data set, the numbers were as follows. From the 1934 subnuclear proteins in our data set, 736 (38%) were predicted through homology-based inference (HB), and 1096 (57%) through the SVM Profile Kernel (ML). For 102 proteins (5%), neither HB nor any of the 13 SVMs predicted any nuclear sub-compartment (note: this was only a subset of all prediction mistakes).

For the second prediction task (nuclear-only vs. traveler proteins) the final method combined homology-based inference and machine learning (again a Profile Kernel SVM) essentially in the same straightforward manner: take HB if possible. The final method (also referred to as *LocNuclei*) performed best at the PSI-BLAST *E*-value ≤10^− 5^ reaching an overall performance of Q_2_ = 72 ± 2% (Additional file 1: Figure S1). In detail of the 1098 nuclear proteins in the corresponding data set, 419 proteins (38%) were predicted by homology-based inference, all other 679 (62%) using the SVM Profile Kernel.

### Good predictions also for minority classes

*LocNuclei* distinguished between 13 different nuclear sub-structures. One crucial challenge for predicting many classes was the lack of experimental annotations for the *minority classes*, i.e. those with fewer known proteins. For instance, the SVM had to generalize from only 14 proteins in the *spindle apparatus* and from only 13 in the *perinucleolar sub-structure* (Additional file 1: Table S1). Nevertheless, *LocNuclei* succeeded in predicting for minority classes, e.g. 8 of the 14 samples for *spindle apparatus* were predicted correctly. The worst performance was observed for the *Cajal body*: 10 of the 42 predicted in this sub-structure were correctly predicted, while an equal number of 10 proteins were mis-predicted to be in the nucleoplasm (Table 1). All these ten mis-predictions originated from the SVM prediction. Using exclusively homology-based inference correctly predicted 8 of 42 Cajal bodies and no misclassification to nucleoplasm would occur (Additional file 1: Table S2).

**Table 1:** LocNuclei confusion matrix for 13 nuclear sub-structures

Observed:- > Predicted:	Chromatin	Nucleolus	Nuclear speckle	PML body	Nuclear lamina	Nuclear matrix	Nuclear envelope	Cajal body	Nuclear pore complex	Nucleoplasm	Kinetochore	Spindle apparatus	Perinucleolar	SUM predicted
Chromatin	**506**	32	11	7	0	6	3	1	1	4	6	1	1	579
Nucleolus	49	**461**	29	11	4	12	3	6	2	3	2	1	2	585
Nuclear speckle	14	19	**153**	2	2	4	1	1	0	2	1	0	0	199
PML body	12	13	9	**38**	2	3	2	1	1	0	1	0	0	82
Nuclear lamina	5	6	3	2	**41**	3	7	0	2	0	1	0	0	70
Nuclear matrix	7	9	5	4	2	**25**	1	0	0	1	0	0	0	54
Nuclear envelope	4	4	1	0	3	1	**34**	0	6	0	1	0	0	54
Cajal body	3	5	2	0	1	1	0	**10**	0	1	0	0	0	23
Nuclear pore complex	4	4	1	2	3	0	6	0	**15**	0	1	0	0	36
Nucleoplasm	39	38	30	11	7	8	5	10	3	**13**	2	1	2	169
Kinetochore	4	5	0	1	1	0	1	1	2	2	**5**	0	0	22
Spindle apparatus	32	27	19	8	10	9	7	5	1	1	1	**8**	1	129
Perinucleolar	0	3	0	0	0	0	0	1	0	0	0	0	**4**	8
None	18	27	29	9	4	2	2	6	1	2	4	3	3	110
% observed	33	31	14	4	4	3	3	2	2	1	1	1	1	
SUM observed	697	653	292	95	80	74	72	42	34	29	25	14	13	

The confusion matrix for LocNuclei predictions on the development set with the columns showing the number of observed and the rows the number of predicted proteins (as shown by the sums provided in the last column and the last row). Correct predictions shown on the diagonal are highlighted in bold. The sub-structures are sorted by the number of available annotations (smallest classes at the bottom/right). The dataset was highly imbalanced in the sub-structures, e.g. only 27 proteins were annotated in spindle apparatus and perinucleolar and the smallest seven of the 13 classes together accounted for only 11% of all annotated, unique proteins (percentage values are given in the row “% observed”). Performance was largely proportional to the class size, i.e. worse for smaller. Nevertheless, LocNuclei succeeded to predict compartments with only a few samples in the training set, e.g. 8 of the 14 proteins located in the Spindle apparatus are correctly predicted

### Reliability index allows focus on best predictions

**Fig. 2:**
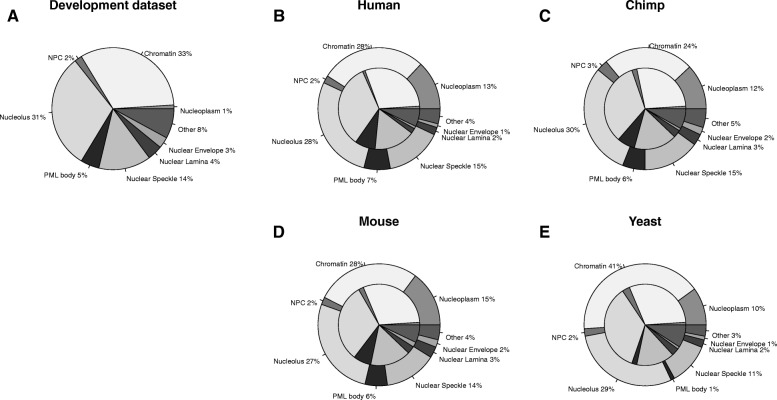
Highly reliable predictions more accurate. The reliability index (RI, x-axis) of *LocNuclei* scaled between 0 (unreliable) and 100 (reliable). It related the prediction strength to the performance. The data for this figure were binned in intervals of 20. Each point reflected the cumulative performance, i.e. we computed accuracy (Q_13_ and Q_2_) and coverage (percentage of proteins for which predictions were made above given RI). **a** For the prediction of 13 nuclear sub-structures, 19% of all proteins were predicted at RI > 60 (point marked by dotted lines). For this top 19%, accuracy rose from the average Q13 = 62% (indicated by leftmost black point) to 75% (point marked by dotted lines). For our data set, RI < 20 did not correlate with accuracy. **b** For the prediction of traveler proteins, 29% of all proteins were predicted at RI > 60 (part B, point marked by dotted lines) with Q2 = 78% (point marked by dotted lines, improving over the average of 72% by six percentage points)

For each prediction, *LocNuclei* also provides a reliability index (RI) that reflects the prediction strength. The RI was scaled to values between 0 (uncertain prediction) and 100 (reliable prediction). Although the RI scaling did not correlate with performance throughout its entire interval, it enables users to focus on reliably predicted proteins: e.g. of the 25% most strongly predicted proteins, 76% were correctly predicted (RI > 50, Fig. 2a: dashed lines).

For the second prediction task (traveler), the reliability index correlated slightly better with performance in the sense that with increasing RI Q_2_ increased (albeit not significantly above values of RI = 50, Fig. 2b). For RI > 50, *LocNuclei* predicted for 45% of the proteins and 77% of these were predicted correctly (Fig. 2b: dashed line).

### Performance of LocNuclei confirmed for independent data set of novel proteins

The only method for predicting nuclear sub-structures available during the development of our new method was NSort [12]. Comparing the two methods back-to-back using values published was meaningless due to the differences in data sets. Being no longer available, *NSort* could not be run on new data. Thus, the only meaningful benchmark required training and testing *LocNuclei* on the sets used for *NSort*. Towards this end, we downloaded the *NSort* data set from http://bioinf.scmb.uq.edu.au:8080/nsort/db and split into five subsets, trained on four and tested on the remaining one. These sets were rotated five times, so that each protein in the NSort set was tested exactly once. The area under the ROC curve (AUC) calculated from the test proteins proxied performance for comparability. For training, we used the same parameters as for the original method. The data set of *NSort* contained proteins from eight sub-nuclear localizations; *LocNuclei-NSort* performed equally well as or even better than *NSort* except for proteins located in the perinucleolar (Table 2). Comparing the original version of *LocNuclei* predicting 13 classes with the version re-trained on eight using the *NSort* data set using common proteins showed that *LocNuclei* performed on average equally well (Additional file 1: Table S3).

**Table 2:** Comparison between LocNuclei and NSort

Sub-nuclear compartment	Number of proteins	AUC NSort	AUC LocNuclei-NSort
Perinucleolar	24	0.80 ± 0.05	0.73 ± 0.03
Cajal body	49	0.60 ± 0.03	0.62 ± 0.02
Nuclear pore complex	51	0.79 ± 0.05	0.88 ± 0.02
Nuclear lamina	77	0.70 ± 0.01	0.82 ± 0.01
PML bodies	91	0.77 ± 0.03	0.75 ± 0.01
Chromatin	323	0.71 ± 0.01	0.78 ± 0.01
Nuclear speckle	403	0.71 ± 0.01	0.77 ± 0.01
Nucleolus	598	0.60 ± 0.01	0.72 ± 0.01
Sum/Mean	1285	0.71 ± 0.03	0.76 ± 0.02

For this comparison, LocNuclei was re-trained using the development data of NSort, comprising 1285 sequence-unique proteins annotated in eight sub-nuclear localization classes. On proteins from all eight classes, LocNuclei performed equally well as or better than NSort except for proteins located in the perinucleolar. The overall cross-validated AUC of LocNuclei was 0.76 compared to 0.71 for NSort. The values for NSort were taken from its publication [12]

### Spectra of sub-nuclear distributions predicted between organisms differ

After completing the development, *LocNuclei* was applied to predicting the nuclear sub-structures for entire proteome in *Homo sapiens* (human), *Pan troglodytes* (chimp), *Mus musculus* (mouse) and *Saccharomyces cerevisiae* (baker’s yeast). Human, mouse and baker’s yeast contribute the most proteins to the development set (341, 961, and 101, respectively). Chimp was only chosen because we expect it to be very close to human. LocTree3 [13] provided the whole proteome predictions for all four organisms (https://rostlab.org/services/loctree3/proteomes). All proteins predicted as nuclear and nuclear membrane were used. The resulting datasets contained 6123 proteins for human, 7358 proteins for mouse, 4761 proteins for chimp and 2107 for yeast.

**Fig. 3:**
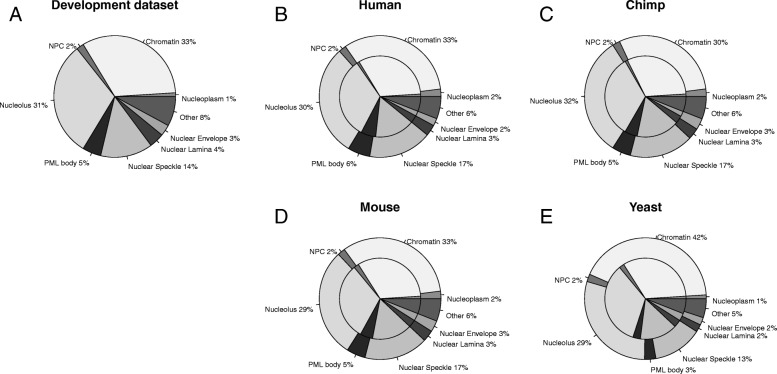
Composition of sub-nuclear compartments in **a** LocNuclei's development set, **b** the human, **c** chimp, **d** mouse and **e** yeast proteome. Proteomes show in B-E are corrected for prediction bias. Most machine learning tools have a prediction bias leading to wrong estimates of distributions. Using the confusion matrix of the development set, this bias can be corrected leading to more realistic estimates of the distribution [14]. When applying this correction to the sub-nuclear predictions for human, chimp, mouse and yeast, the distributions for human, chimp and mouse look more similar to the development set. For all four organisms, the fraction of proteins annotated to the nucleoplasm decreases after the correction

Most machine learning tools have some kind of prediction bias overestimating some classes while underestimating others. To correct for this bias, it was proposed to use the confusion matrix of the tool based on the development set [14]. This leads to an estimation of the overall class distribution that is closer to the truth than the actual predicted distribution. The compositions of the predicted sub-nuclear compartments, i.e. the sub-nuclear spectra were very similar for all organisms for the part only using homology inference (Fig. 3b, c, d and e inner circles). When applying the bias correction to the whole dataset, the composition for human, mouse and chimp remained similar (Fig. 3b, c and d). For human, chimp and mouse, the distributions were also close to the one of the development set (Fig. 3). Only the distribution for yeast differed with a higher number of proteins localized to the chromatin than for the other organisms (Fig. 3e). For all organisms, most proteins were predicted to be either in the chromatin or in the nucleolus (Fig. 3b, c, d and e). Chromatin is a structure built from the interaction with DNA and its role is the maintenance of DNA and the regulation of its transcription. It is known that many proteins that compose the chromatin are exchanged with other sub-nuclear compartments, such as the nucleolus [15, 16].

Using the given distribution, we can also calculate the Euclidean distance between these distributions and use them as a proxy for the distance between the organisms. In our lab, it has been shown that the simple predicted location spectra using all subcellular localizations capture evolutionary aspects of cross-species comparisons [17]. Applying the same concept to the subnuclear location spectra suggested yeast to be most distant from human, chimp and mouse while the distance between human and mouse was smaller than that between human and chimp (Table 3). These differences were statistically significant. If we consider de novo prediction and homology-based inference separately, the relation between organisms based on the distances of the subnuclear location spectra did not change for de novo prediction while the location spectra predicted through homology-based inference reflected the expected relation, i.e. human appeared closest to chimp and most distant to yeast (Additional file 1: Table S4).

**Table 3:** Euclidean distance between organisms based on predicted subnuclear location spectra

Overall				
	Human	Chimp	Mouse	Yeast
Human	0	4.0 ± 0.6	1.8 ± 0.3	9.7 ± 0.7
Chimp	4.0 ± 0.6	0	4.0 ± 0.5	12.7 ± 0.8
Mouse	1.8 ± 0.3	4.0 ± 0.5	0	9.9 ± 0.7
Yeast	9.7 ± 0.7	12.7 ± 0.8	9.9 ± 0.7	0

We calculate the Euclidean distance between predicted subnuclear location spectra and use that distance as proxy to identify evolutionary relationships. As expected, yeast is most distant from the other organisms. However, according to the subnuclear location spectra, human is closer to mouse than to chimp which is opposite what we would expect from known evolutionary relationships. Predicted subnuclear location spectra help in identifying certain aspects of evolution while they cannot capture all evolutionary relations in detail

### Predictions for homologous protein pairs from different organisms agreed

For a more fine-grained analysis, we also compared predictions for pairs of homologous proteins. For each of the six possible organism pairings, we identified all pairs of homologous proteins in the same way used for LocNuclei (PSI-BLAST at *E*-Values≤ 10^-20^). The resulting number of homologous protein pairs mirrored the distance between the predicted subnuclear location spectra (Table 3) for these organisms: For 70% of the human nuclear proteins, we found a homologous protein in mouse (Table 4); the distance between these two organisms based on the location spectra was also the smallest. For yeast, which was most distant to the other organisms, we only found homologous proteins for 20–23% of the proteins (Table 4). For all organism pairs, most protein pairs were predicted by homology-based inference (Table 4, third column). For only a few protein pairs (2% or 6%), one of the proteins was predicted using homology-based inference while the other one is predicted de novo (Table 4, fourth column).

**Table 4:** Homologous protein pairs between four different organisms

	Overall	Both HB	HB/SVM	SVM	% of proteins (organism1)	% of proteins (organism2)
Human/Chimp	3663	2510 (68%)	58 (2%)	1095 (30%)	60%	62%
Human/Mouse	4316	2609 (60%)	67 (2%)	1640 (38%)	70%	50%
Human/Yeast	809	638 (79%)	50 (6%)	121 (15%)	13%	21%
Chimp/Mouse	4041	2667 (66%)	65 (2%)	1309 (32%)	65%	55%
Chimp/Yeast	776	608 (78%)	42 (6%)	126 (16%)	16%	20%
Mouse/Yeast	973	742 (76%)	50 (5%)	181 (19%)	13%	23%

We identified pairs of homologous proteins between human, chimp, mouse, and yeast. The second column in the table gives the overall numbers of pairs for these two organisms, the next three columns refer to pairs of proteins where both were predicted using homology-based inference, one was predicted with homology-based inference and the other one de novo, and both were predicted de novo. The last two columns give the percentage of proteins in the respective organisms for which a homolog was found

**Fig. 4:**
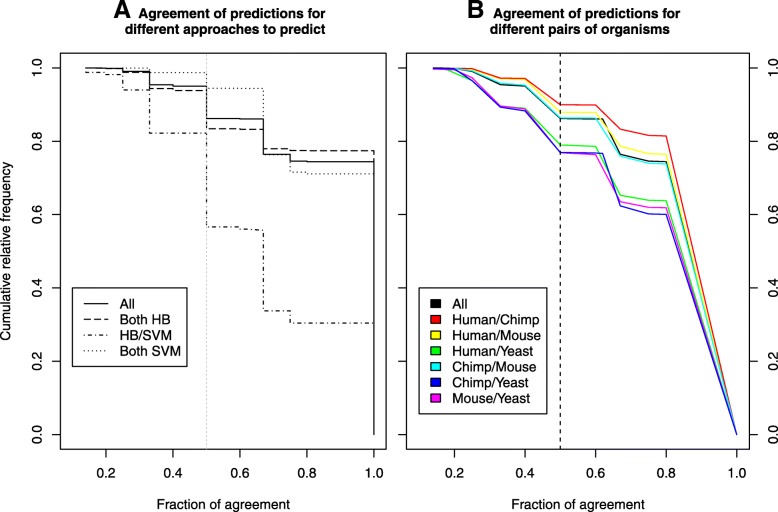
Agreement for predictions of homologous protein pairs. 95% of all pairs of homologous proteins agreed to a score ≥ 0.5 (Eq. 5); 74% reached full agreement (score = 1; black lines in A and B). **a** If both proteins were predicted using the same approach (either homology-based inference or de novo), the fraction of agreement was very similar. Predictions disagreed more when the proteins were predicted by different approaches. **b** Predictions for yeast proteins and homologs in any other organism disagreed more than for other pairs of organisms

For pairs of homologous proteins, we expect similar predictions from *LocNuclei*. The similarity in predictions between two proteins was measured through the fraction of agreement (Eq. 5; note: for some proteins more than one class was predicted). For almost three fourth (74%) of all protein pairs this agreement was 1, i.e. all classes were predicted identically; while for over 95% of the pairs the agreement scores were ≥ 0.5 (Fig. 4a). Surprisingly, the agreement was essentially the same if both proteins were predicted by homology-based inference (HB) and de novo by machine learning (ML, Fig. 4a dashed and dotted lines). Only for mixed protein pairs (one predicted by HB, the other by ML) predictions agreed much less (Fig. 4a: lowest line with dots and dashes). However, these pairs constituted a small fraction of the overall set of protein pairs (Table 4, fourth column; 2% of all pairs of homologous proteins).

For the set of four model organisms (human, chimp, mouse, and yeast), predictions for homologous proteins agreed most between human and chimp, slightly less between human-mouse or chimp-mouse, and least for human-yeast, mouse-yeast and chimp-yeast (Fig. 4b). As yeast is the most distant from the other organisms, it is most likely that yeast proteins have different sub-nuclear locations although related in evolution.

Overall, homologous protein pairs, obviously share sub-nuclear locations, otherwise, homology-based inference would not work for our predictions. Nevertheless, for some protein pairs predictions agreed poorly, with the minimal agreement of 0.14 and with 21 protein pairs having agreement ≤0.2. Of these 21 proteins, only eight include proteins from yeast; most (15) include a protein from mouse. The agreement score inversely correlated with the number of compartments predicted differentially between the two organisms. For instance, for the four worst predictions (agreement = 0.14), seven compartments were predicted for one organism, but only two for the other. The second protein with two predicted compartments was always the probable E3 ubiquitin-protein ligase HUL4 from yeast (Uniprot identifier P40985) while the other four proteins seemed to belong to the same family. Three proteins were from mouse (genes Herc6, Herc4, and Herc3; Uniprot identifier F2Z461, Q6PAV2, A6H6S0) and the fourth protein was from the chimp gene HERC6 (Uniprot identifier H2QPV8).

### GO enrichment of sub-nuclear predictions

Subcellular localization is one aspect of protein function. Thus, the Gene Ontology (GO) [18, 19] reserves one of its three ontologies for function to *Cellular Component* (the other two being *Molecular Function* and *Biological Process)*. This does not strictly imply that the *LocNuclei* predictions correlate with function as described by the BFO (Biological Process Ontology of GO). Nevertheless, we hypothesized that there is a correlation.

To address this hypothesis, we performed a GO enrichment analysis of terms from the BFO for the human nuclear proteins predicted by *LocNuclei*. Experimental annotations were available for 4667 of the 5088 (92%) predicted human nuclear proteins. For each of the 13 nuclear sub-structures, we identified the BFO-terms enriched at highest statistical significance (*p*-value< 0.01, Additional file 1: Table S5). Only for 10 of the 13, more than 10 BFO terms reached *p*-values< 0.01 (only 2 for peri-nucleolar, only one for nucleoplasm, and none for the spindle apparatus, Additional file 1: Table S5).

The *nucleolus* is involved in ribosomal biogenesis [20] and *LocNuclei* predicted 1856 of the 5088 (36%) human nuclear proteins at the nucleolus. For these proteins, the BFO terms “rRNA processing”, “rRNA metabolic process”, “RNA modification” and “ribonucleoprotein complex biogenesis” were prominent amongst the ten terms with the lowest *p*-value (highest significance, Additional file 1: Table S5). *Chromatin* packages DNA and regulates the access of DNA-binding proteins [21]. For the 1901 proteins predicted to locate to the chromatin (37% of all nuclear proteins) enriched BFO terms included “chromatin organization”, “regulation of RNA biosynthetic process” and “regulation of transcription, DNA-templated” (Additional file 1: Table S5). *Kinetochores* are protein complexes that form when a cell divides; they are located at the centromere and attach the duplicated chromosomes to the mitotic spindle to allow their separation [2]. Only 42 proteins were predicted to locate to the *kinetochores*. For these proteins enriched BFO terms included “cell division”, “chromosome segregation”, and “attachment of spindle microtubules to kinetochores” (Additional file 1: Table S5). Although only few (42) proteins were predicted for kinetochores, the GO enrichment analysis revealed a clear link between the predicted localization and function. Overall, the results of the enrichment analysis for nucleolus, chromatin and kinetochore clearly supported the hypothesis that the predicted sub-nuclear location provided important new evidence for inferring protein function. The results for other compartments such as *nuclear pore complex* and *nuclear envelope* also supported the hypothesis (Additional file 1: Table S5).

For other sub-structures, the signal was less clear. One extreme negative example was the *spindle apparatus* for which not a single BFO term was enriched statistically significantly. The problem might have been that only 13 proteins were predicted in this sub-structure (Additional file 1: Table S5) limiting the power of an enrichment analysis. Another extreme example was the *nucleoplasm* for which 852 proteins (17% of all) were predicted but only one BFO term was statistically significant (namely *Keratinization*, Additional file 1: Table S5). The problem here might have originated from the diversity of this sub-structure that might also result in many prediction mistakes (Table 1). The third sub-structure for which we found fewer than 10 BFO terms enriched at *P*-values< 10^− 2^ was the *perinucleolar* (two terms enriched in 33 predicted proteins, Additional file 1: Table S5). For another sub-structure full of a variety of very different proteins [22], the *PML bodies*, our hypothesis was also not supported making it difficult to clearly infer function from enrichment of GO terms.

Performing the same analysis for traveler proteins shows that the most significantly enriched BFO terms for traveler proteins are all associated with transport and localization (Additional file 1: Table S5) suggesting that traveler proteins travel in and out of the nucleus to transport molecules and guide protein localization. Less, but still significantly enriched terms also include involvement in signal transduction (e.g. GO35556 – intracellular signal transduction, GO0023051 – regulation of signaling, or GO0010646 – regulation of cell communication).

## Protein-protein interactions (PPI) related to predicted sub-nuclear localizations

Another way to proxy biological processes is through monitoring physical protein-protein interactions (PPIs^1^) [23]. In analogy to the BFO enrichment analysis, we tested whether or not proteins predicted in nuclear sub-structures by LocNuclei contained information about PPIs. More explicitly, we analyzed whether the experimentally annotated PPIs are overrepresented for certain compartments. Overrepresentation is described by the odds ratio that sets the number of observed PPIs between proteins in two compartments (or the same one) into relation with the expected number of PPIs between these compartments. An odds ratio below 1 indicates less PPIs than expected, 1 indicates as many PPIs as expected and values above 1 indicate more PPIs than expected.

**Fig. 5:**
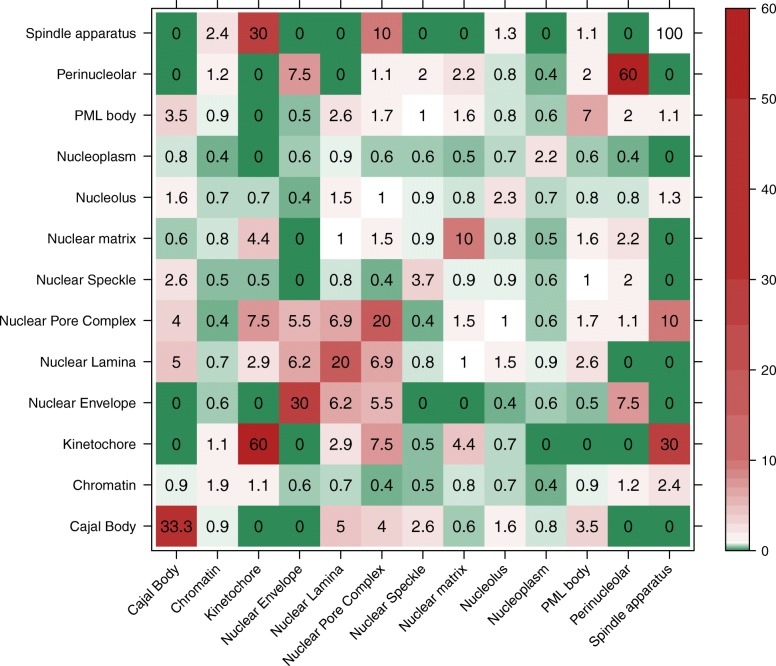
Odds ratio of protein-protein interactions (PPIs) in the human nuclear proteome. The heatmap shows the odds ratio of PPIs within and between 13 sub-nuclear compartments. The experimentally annotated PPIs were extracted from mentha [46], the Integrated Interactions Database (iid) [47], the Human Reference Protein Interactome Mapping Project (HuRI) [48] (data gathered by the Center for Cancer Systems Biology at the Dana-Farber Cancer Institute and supported by the National Human Genome Research Institute of NIH, the Ellison Foundation, Boston, MA and the Dana-Farber Cancer Institute Strategic Initiative, accessed on 14-02-2018), HINT [49], iRefIndex [50], InBio Map [51] and mapped to those in human proteins of 13 predicted sub-nuclear compartments. Values below 1 (depicted by green colors) indicate less PPIs between proteins in these compartments than expected, values equal to 0 (depicted by white) indicate as many PPIs as expected and values above 1 (depicted by red colors) indicate more PPIs than expected. PPIs are observed more often than expected within all compartments and also between proteins in the Spindle apparatus and Kinetochore as well as proteins in the Nuclear Pore Complex, Nuclear Lamina and Nuclear Envelope which can be explained by the shared functionality of the different compartments

Toward this end, the set of human proteins with predicted sub-nuclear localizations were mapped to a dataset of binary, direct interactions from multiple sources used in a different context by our group [24]. In this set, more PPIs than expected are observed within all compartments with especially high values for PPIs between proteins within the kinetochore and the spindle apparatus (Fig. 5) indicating that the formation of compartments and the functionality of proteins performed in these compartments highly relies on interaction between proteins. PPIs between proteins in different compartments are either underrepresented or close to expected except for interactions between proteins in the kinetochore and the spindle apparatus as well as between proteins in the nuclear pore complex, the nuclear lamina and the nuclear envelope (Fig. 5).

Another way to analyze PPIs within nuclear proteins is to compare them to proteins outside the nucleus. To do so, we constructed a PPI network from the human PPI data with proteins being the nodes and an edge drawn between proteins when they interact. The network consists of 15,634 nodes in 569 connected components. Only 142 of these components consist of more than one node. Of the 15,634 proteins in the network, 2037 are solely located in the nucleus, 1283 are traveler proteins travelling between the nucleus and other compartments and 12,314 are proteins located outside the nucleus. On average, nuclear proteins in this network have an average degree of 18 for non-traveler and of 20 for traveler while non-nuclear proteins only have a degree of 10. Considering only the largest connected component with 14,875 does not significantly change the average degree. So, on average nuclear proteins have a higher degree, i.e. they are interacting with more other proteins, than non-nuclear proteins. Also, traveler proteins have a slightly higher degree than non-traveler proteins indicating that they need to interact with other proteins to move in and out of the nucleus. Also, most of the nuclear proteins (97%) are located in the largest connected component, so they are an important part of the PPI network.

## Discussion

*LocNuclei* predicts sub-nuclear localization at a high accuracy. It combines homology-based inference and de novo prediction to achieve the highest performance. The relatively conservative threshold at which the combination was best (Fig. 1: *E*-value ≤ 10^-20^) was surprising due to its extremity (e.g. thresholds down to *E*-values ≤ 10^− 3^ are often used to infer functional similarity), and due to the fact that the performance for lower values was still higher than that of the machine learning (Fig. 1: “Homology (with hit)” vs. SVM). In fact, the curve remained numerically higher down to *E*-values≤10^− 5^ (straight gray line at Q_13_ = 59% vs. dark line in Fig. 1). Given the simple algorithm for the combination of homology-based inference (HB) and machine learning (ML) (if ∃ HB, take HB, else take ML) the combined algorithm could never be worse than its constituents (HB&ML > max (HB,ML)). Thus, the optimality in Q_13_ of a conservative threshold suggested that some of the proteins for which HB was available were also predicted above average for ML. Conversely, the cases added at lower thresholds of HB were predicted better by ML than by HB thereby reducing performance by choosing HB over ML (Fig. 1 threshold between 10^−20^ and 10^− 5^ all have HB above the ML performance).

Trained on the *NSort* training data, *LocNuclei-NSort* outperforms *NSort*, a predictor for eight sub-nuclear localization classes. On the one hand, it appeared that *LocNuclei* did not gain much from more recent data. On the other hand, it appeared not to have lost from distinguishing more classes.

Spectra for subnuclear compartments calculated from the distribution of the actual predictions show that none of the spectra for human, chimp, mouse, or yeast resembled that for the development set (Additional file 1: Figure S2A) suggesting that the new method was not completely biased by its development set and could discover important aspects in the nuclear proteomes of human, chimp, mouse, and yeast. The biggest difference was for the nucleoplasm for which a much large fraction was predicted in all organisms than in the development set. Since the fraction of proteins predicted in the nucleoplasm decreases when applying a correction (Fig. 3), this suggests a bias in the prediction towards overestimating the number of proteins located to that compartment.

The Euclidean distance between subnuclear location spectra is used to discover evolutionary relationships between organisms. However, the discovered relations between human, chimp, mouse, and yeast are not all as expected (e.g. human closest to mouse instead of chimp). So, while the comparison of subnuclear location spectra can reveal some insights into the evolutionary relationship between organisms (e.g. human, chimp and mouse closer to each other than to yeast), not all evolutionary aspects can be uncovered completely. Either the subnuclear spectra do not carry enough information to capture evolutionary relationships between these organisms fully or the de novo method makes too many mistakes when predicting subnuclear compartments so that not enough information is left to reconstruct the evolutionary relationships correctly.

For pairs of homologous proteins, the predicted sub-nuclear compartments often agree. However, there are some pairs where the predictions are very different, especially in terms of number of predicted compartments. We could not find any evidence in public databases or the literature that the difference in predicted compartments for these protein pairs is reasonable. Therefore, the major reason for a disagreement in predictions between homologous proteins seems to be that *LocNuclei* predicts too many compartments for certain proteins. In fact, this observation is also true for the development set: For 34% of the proteins, the correct number of compartments is predicted, for 61%, at least one compartment more is predicted than annotated, and for 44%, even at least two compartments more are predicted than annotated.

Predicted subnuclear compartments can reveal insights into a protein’s functionality. GO enrichment analysis revealed a clear link between the predicted localization and function for many compartments (e.g. nucleolus and kinetochores) while the signal was less clear for other compartments (e.g. nucleoplasm and PML bodies). Overall, the inference of function (as proxied by BFO) from LocNuclei predictions worked best for compartments with a stable structure and a clearly defined function.

Monitoring PPIs provides another way to proxy biological processes. As expected, the number of PPIs between proteins within the same compartment is always high while the number of PPIs between proteins in different compartments is much lower. There are only a few exceptions (PPIs between kinetochores and spindle apparatus, and between nuclear pore complex, nuclear lamina, and nuclear envelope occur more often than expected) and these ones can be explained by the shared functionality of the proteins in these compartments. The kinetochore is responsible for attaching the duplicated chromosomes to the spindle apparatus [2] making PPIs between these two compartments inevitable for proper functionality. Nuclear pore complex, nuclear lamina and nuclear envelope are all part of the nuclear membrane suggesting that interactions between proteins of these compartments are needed for stability and proper functionality of the nuclear membrane. As the GO enrichment analysis, the analysis of PPIs between sub-nuclear human proteins showed that the predicted nuclear sub-structures related to the expected functionality of sets of proteins. Therefore, being able to correctly predict subnuclear compartments can help in identifying probable PPIs and functionality.

## Conclusions

*LocNuclei* is an easy-to-use new method predicting sub-nuclear localization; it combined homology-based inference (using PSI-Blast) and de novo prediction (machine learning through an SVM Profile Kernel) to predict the most likely of 13 sub-nuclear compartments in which a nuclear protein functions. It used a similar technology to distinguish between proteins functional only in the nucleus and those also functional in other non-nuclear compartments (dubbed *traveler proteins*). Fivefold stratified cross-validation yielded Q_13_ = 0.62 ± 0.03 (one standard deviation) for the sub-structure prediction and Q_2_ = 0.72 ± 0.02 for the traveling proteins. These high values constituted another example for the scientific merit of the Profile Kernel technology [25].

Six thousand one hundred twenty-three proteins of 20,248 of the human proteins (30%) were predicted by *LocTree3* to be located in the nucleus. Here we introduced a set of new methods, referred to as *LocNuclei* that mapped these proteins onto 13 sub-nuclear structures. Most of the nuclear proteins (57%) were predicted to function in the chromatin or the nucleolus. *LocNuclei* also distinguished between traveler and non-traveler proteins. This method suggested only about one third of all nuclear proteins to also function outside the nucleus.

GeneOntology (GO) enrichment analyses focusing on the BFO (Biological Process Ontology) suggested that BFO terms can be inferred from the predicted sub-nuclear locations, at least for stable localizations with a clearly defined role. By cross-referencing the mapped human nuclear proteome protein-protein interaction (PPI) data, an overrepresentation of interactions of proteins within a compartment as well as between proteins located to the kinetochores and the spindle apparatus or proteins located to the nuclear lamina, nuclear envelope, and nuclear pore complex were observed. Like the BFO enrichment, the PPI enrichment suggested that LocNuclei predictions might help in annotating protein networks.

## Methods

### Data set for development and evaluation

Experimentally annotated nuclear proteins and annotations for their sub-nuclear localization were combined from six databases: HPRD [26], NMPdb [27], NOPdb [28], NPD [29], NSort/DB [30], and Swiss-Prot [31]. These databases differ in some of their annotation terms for sub-nuclear compartments. We “normalized” these differences through a set of 13 distinct keywords describing the sub-nuclear data set (Additional file 1: Table S6).

Of 12,055 proteins experimentally annotated as nuclear, only 3522 (29%) were associated with one or more nuclear sub-structure. *UniqueProt* [32] generated a non-redundant subset for these by only accepting pairs with HVAL< 20 [33, 34] (implying less than 40% pairwise sequence identity for alignments over 250 residues). At lower HVALs, the data set became too small for meaningful performance estimates. The final sequence-unique sub-nuclear set comprised 1934 proteins (Additional file 1: Table S1).

Four thousand seven hundred twenty-two of the same 12,055 nuclear proteins were also annotated in at least one other non-nuclear sub-cellular compartment (e.g. the mitochondria). The complete set of 12,055 nuclear proteins was redundancy-reduced at HVAL< 0 yielding 1098 sequence-unique proteins, of which 559 (51%) were annotated to exclusively localize to the nucleus, 539 (49%) to be in the nucleus and some other compartment.

The resulting prediction method was trained to differentiate between (i) proteins localized solely to the nucleus and proteins localized to the nucleus and other sub-cellular compartments (traveler proteins), as well as between (ii) proteins of the 13 sub-nuclear localization classes.

### Prediction methods

*LocNuclei* combined homology-based inference and machine learning-based *de novo* predictions in the same way LocTree3 [13] does: if a sequence similar to a protein of experimentally known localization is available that annotation is transferred, if not, the machine learning-based prediction is returned. Stratified fivefold cross-validation was used to determine all parameters and to assess the performance. In a stratified cross-validation, the distribution of classes is approximately equal in every subset [35].

### Homology-based inference

PSI-BLAST [36] alignments are used to transfer annotations by homology. For all proteins of known localization, PSI-BLAST profiles were generated with two iterations and E-value ≤ 10^− 3^ using an 80% non-redundant database combining UniProt [37] and PDB [38]. These profiles were then aligned at E-value ≤ 10^− 20^(for prediction of subnuclear compartments) or ≤ 10^− 5^ (for prediction of traveler proteins) against non-redundant proteins in the development set. For performance estimates, PSI-BLAST self-hits were excluded. The annotation from the hit with the highest pairwise sequence identity of all retrieved alignments was transferred to the query protein.

#### De novo prediction

The SVM [39] implementation of LibSVM [40] and the Profile Kernel Function [25, 41] was used to train 13 different SVM classifiers to predict 13 sub-nuclear localizations, where each classifier was trained to discriminate between all the proteins in one particular nuclear sub-structure and all proteins in any of the other 12 nuclear sub-structures. Another profile kernel SVM learned to distinguish between proteins exclusively observed in the nucleus and those observed in the nucleus and other sub-cellular compartments (referred to as *traveler proteins*)*.*

The Profile Kernel algorithm maps each evolutionary profile to a 20^k^-dimensional vector of integers. Each dimension represents one *k-mer*, a string of *k* consecutive residues and a particular value gives the number of times this *k-mer* is conserved in an evolutionary profile (multiple sequence alignment). Conservation is calculated as the sum of substitution scores for each residue in the *k-mer* and has to fall below a certain threshold *σ* [25, 41]*. σ* and *k* are user defined parameters that we optimized during training. For the SVMs, we focused on optimizing C, the penalty parameter of the error term, and tol, the tolerance for the stopping criterion. For each Profile Kernel SVM, we optimized these four parameters independently. Also, class weights inversely proportional to class frequencies in the input data were applied for the subnuclear prediction to correct for class imbalance. The traveler dataset was almost balanced; thus, we did not apply class weights for this prediction task. All chosen parameter settings for the 14 different SVMs are listed *in* Additional file 1*:* Table S7.

#### Reliability index (RI)

Prediction strength correlated with performance (Fig. 2) allowing users to focus on more reliable new predictions through a reliability index (RI) ranging from 0 (weak prediction) to 100 (confident prediction). For the homology-based inference, the percentage pairwise sequence identity (PIDE) from PSI-BLAST was used to define the RI (RI = int(10*(PIDE-20)/8)). To convert the raw SVM score to a reliability index, this score is normally transferred to a probability using Platt scaling [42]. However, the implementation of Platt scaling in LibSVM [40] failed for our dataset. Typically, SVM scores > 0 should give probability values > 0.5. For our dataset, this was only observed for the prediction of some sub-structures (classes). For others, Platt scaling transferred the scores to probabilities < < 0.5. Therefore, we had to renormalize the raw SVM scores (Eq. *1*) as follows:

1 $$ \mathrm{R}{\mathrm{I}}_{\mathrm{svm}}={\mathrm{raw}}_{\mathrm{svm}}\bullet \frac{100}{\max \left(\mathrm{ra}{\mathrm{w}}_{\mathrm{svm}}\right)} $$

### Performance evaluation

The performance of *LocNuclei* was assessed through standard measures. For each localization class, every prediction can be classified as either true positive (TP, the sample is predicted and observed in this class), false positive (FP, the sample is predicted in this class, but observed in another), false negative (FN, the sample is predicted not to be in this class but observed in it) and true negative (TN, the sample is predicted and observed in another class). From this classification, the overall accuracy follows:

2 $$ Q(n)=100\bullet \frac{\sum_{i-1}^n number\ of\ proteins\ correctly\ predicted\ in\ class\ i}{\sum_{i=1}^n total\ number\ of\ proteins\ observed\ in\ class\ i} $$

with n as the number of localization classes (here: 13). To simplify, this measure calculates the total number of correct predictions divided by the total number of proteins in the test set.

The receiver operating characteristic (ROC) curve and the derived area under the curve (AUC) are combined performance measures connecting true positive rate (TPR, Eq. 3) and false positive rate (FPR, Eq. 4) [43]. The ROC-curve shows FPR versus TPR.

3 $$ TPR=100\bullet \frac{TP}{TP+ FN} $$

4 $$ FPR=100-100\bullet \frac{TN}{TN+ FP} $$

The curve is often simplified into a single number, the Area Under the Curve (AUC) [43].

### Comparison of LocNuclei predictions between proteins

*LocNuclei* might predict more than one sub-nuclear compartment for a particular protein. This implies that the comparison of predictions between, e.g. two similar/homologous proteins requires the introduction of additional parameters. Toward this end, we used the fraction of agreement in two predictions A^n^ and B^m^ defined as follows:

5 $$ agree\left({A}^n,{B}^m\right)=\frac{1}{n}\bullet {\sum}_{i=1}^n{z}_i,{z}_i=\left\{\begin{array}{c}1, if\ {a}_i\in \left({b}_1,\dots, {b}_m\right)\\ {}0, otherwise\ \end{array}\right.\  and\ n\ge m\ w.l.o.g. $$

where A and B are two proteins and n and m are the number of predicted compartments for A and B, respectively. In the limit of a single prediction per protein, this agreement is identical to the percentage of correct predictions; in the limit of predicting all sub-compartments for one protein, the value falls below random (1/13 which is lower than random given the difference in the size distribution of the 13 compartments).

### GO enrichment analysis

Gene Ontology (GO) [18, 19] provides a controlled vocabulary (GO terms) of annotated functions for a protein. It consists of three separate ontologies: “Biological Process”, “Molecular Function” and “Cellular Compartment”. To analyze whether certain GO terms are statistically enriched for proteins annotated in a particular nuclear sub-structure, we used the webserver *GOrilla* (http://cbl-gorilla.cs.technion.ac.il/) [44]. GOrilla analyzes the enrichment of a certain set of proteins through a hypergeometric distribution. It compares the number of known experimental annotations of a GO term in all proteins within a compartment (positive class) and those in all proteins not in the compartment (negative class). The resulting p-value gives the probability to observe the given annotations under the assumption that the annotations for proteins from both classes do not differ. A small p-value indicates that this assumption is not true and that the corresponding GO term is overrepresented in the positive class. GOrilla also offers correction for multiple testing by giving a p-value adjusted using the Benjamini-Hochberg method [45]. We only considered the adjusted p-value when analyzing the significance of results. We considered all terms with p-values < 0.01 as significantly enriched in the positive. The GO enrichment analysis was carried out exclusively for GO ontology “biological process”.

### Protein-protein interactions (PPI) for nuclear proteins

To analyze the map between nuclear sub-structures and protein-protein interactions (PPIs) in human proteins, we merged a dataset containing information from six original resources, namely: (1) mentha [46], (2) the Integrated Interactions Database (iid) [47], (3) the Human Reference Protein Interactome Mapping Project (HuRI) [48] (data gathered by the Center for Cancer Systems Biology at the Dana-Farber Cancer Institute and supported by the National Human Genome Research Institute of NIH, the Ellison Foundation, Boston, MA and the Dana-Farber Cancer Institute Strategic Initiative, accessed on 14-02-2018), (4) HINT [49], (5) iRefIndex [50], and from (6) InBio Map [51]. For each database, only binary, direct interactions were considered (often also referred to as transient physical interactions), i.e. we excluded associations. Furthermore, only interactions determined by an experiment and validated by a yeast two-hybrid (Y2H) experiment or interactions supported by two independent Pubmed IDs were considered.

To analyze whether proteins between or within a compartment interact more often than we would expect, we calculate an odds ratio for an interaction to happen between compartment *i* and *j* (Eq. 5).

6 $$ odds\left({PPI}_{ij}\right)=\frac{num_{obs}\left({PPI}_{ij}\right)}{num_{exp}\left({PPI}_{ij}\right)} $$

where is the number of expected PPIs between proteins in these compartments and is calculated as

7 $$ {num}_{exp}\left({PPI}_{ij}\right)=\frac{num_{pos}\left({PPI}_{ij}\right)}{\sum_{ij}{num}_{pos}\left({PPI}_{ij}\right)}\bullet {num}_{obs}(PPI) $$

Where *num*_*pos*_ (*PPI*_*ij*_) is the number of possible PPIs between proteins in compartment *i* and *j* in the whole PPI dataset and *num*_*obs*_ (*PPI*) is the overall number of observed PPIs in our data set.

***NSort*** [12] is a framework with eight Bayesian Network-based classifiers that predict protein sub-nuclear localization in eight classes (nucleolus, perinucleolar region, PML bodies, nuclear speckle, Cajal bodies, chromatin and nuclear pore complexes). Each classifier operates from biological features including protein sequence, protein interactions, domain and post-translational modification. Each prediction of *NSort* can be traced back to the feature contributing most to the result. As *NSort* is the only method available to accomplish some of the objectives aimed at by *LocNuclei*, we compared the performance of *LocNuclei* to that of *NSort*.

### Availability

*LocNuclei* is a Python project and is available on GitHub: https://github.com/Rostlab/LocNuclei. The datasets of sub-nuclear and traveler proteins used for development as well as sub-nuclear and traveler predictions for all proteins from the development set are also available. More detailed information on how to run *LocNuclei* is given in the repository.

## Additional file

Additional file 1: **Figure S1.** Effect of E-value thresholds on combined prediction of traveler proteins. **Figure S2.** Composition of sub-nuclear compartments in the human, chimp, mouse and yeast proteome and LocNuclei’s development set. **Table S1.** Composition of the sub-nuclear development set for LocNuclei. **Table S2.** LocNuclei confusion matrix for homology-based inference and machine learning prediction. **Table S3.** Comparison between LocNuclei and LocNuclei-NSort. **Table S4.** Euclidean distance between organisms based on subnuclear location spectra predicted with SVM Profile Kernel (de novo) or homology-based inference. **Table S5.** Top ten statistically enriched GO terms for each sub-nuclear compartment. **Table S6.** Normalization of sub-nuclear localization terms. **Table S7.** Chosen hyperparameters for the 14 different SVM Profile Kernels. (DOCX 43170 kb)

## Abbreviations

AUC: Area under the ROC curve
FPR: False positive rate
GO: Gene Ontology
HB: Homology-based inference
HuRi: Human Reference Protein Interactome Mapping Project
iid: Integrated Interactions Database
ML: Machine learning
NES: Nuclear export signals
NLS: Nuclear localization signals
NPC: Nuclear pore complex
PPI: Protein-protein interaction
ROC: Receiver operating characteristic
SVM: Support vector machine
TPR: True positive rate

## Footnotes

1 Operationally, we defined transient, physical protein-protein interactions (PPI) as cases of two different proteins that come so close in space that they “bind” (physical interaction as opposed to association; closest C-alpha≤6 Å) and that this binding is shorter than the “life”-time of either of the two (transient). This simple definition implies in particular that (i) PPIs are formed only between different proteins, (ii) no transitivity: PPI(A,B) ∩ PPI(B,C) ↛ PPI(A,C), and (iii) no molecular machines: just as most associated proteins do not bind, most members of the same molecular machine, or large physical complex do not bind.

## References

[1] Erhardt M, Adamska I, Franco OL (2010). Plant nuclear proteomics--inside the cell maestro. FEBS J.

[2] Alberts B, Johnson A, Lewis JH, Morgan D (2015). Molecular biology of the cell.

[3] Sampathkumar P, Kim SJ, Upla P, Rice WJ, Phillips J, Timney BL, Pieper U, Bonanno JB, Fernandez-Martinez J, Hakhverdyan Z (2013). Structure, dynamics, evolution, and function of a major scaffold component in the nuclear pore complex. Structure.

[4] Freitas N, Cunha C (2009). Mechanisms and signals for the nuclear import of proteins. Curr Genomics.

[5] Cokol M, Nair R, Rost B (2000). Finding nuclear localisation signals. EMBO Rep.

[6] Bernhofer M, Goldberg T, Wolf S, Ahmed M, Zaugg J, Boden M, Rost B (2018). NLSdb - major update for database of nuclear localization signals and nuclear export signals. Nucleic Acids Res.

[7] Marfori M, Mynott A, Ellis JJ, Mehdi AM, Saunders NF, Curmi PM, Forwood JK, Boden M, Kobe B (2011). Molecular basis for specificity of nuclear import and prediction of nuclear localization. Biochim Biophys Acta.

[8] Carmo-Fonseca M (2002). The contribution of nuclear compartmentalization to gene regulation. Cell.

[9] Chubb JR, Bickmore WA (2003). Considering nuclear compartmentalization in the light of nuclear dynamics. Cell.

[10] Lohrum MA, Ashcroft M, Kubbutat MH, Vousden KH (2000). Identification of a cryptic nucleolar-localization signal in MDM2. Nat Cell Biol.

[11] Eilbracht J, Schmidt-Zachmann MS (2001). Identification of a sequence element directing a protein to nuclear speckles. Proc Natl Acad Sci U S A.

[12] Bauer DC, Willadsen K, Buske FA, Le Cao KA, Bailey TL, Dellaire G, Boden M (2011). Sorting the nuclear proteome. Bioinformatics.

[13] Goldberg T, Hecht M, Hamp T, Karl T, Yachdav G, Ahmed N, Altermann U, Angerer P, Ansorge S, Balasz K (2014). LocTree3 prediction of localization. Nucleic Acids Res.

[14] Marot-Lassauzaie V, Bernhofer M, Rost B. Correcting mistakes in predicting distributions. Bioinformatics. 2018;34(19):3385-3386. 10.1093/bioinformatics/bty346 PMC6157078 29762646

[15] McKeown PC, Shaw PJ (2009). Chromatin: linking structure and function in the nucleolus. Chromosoma.

[16] Bickmore WA, Sutherland HGE (2002). Addressing protein localization within the nucleus. EMBO J.

[17] Marot-Lassauzaie V. Cross-species comparison of protein subcellular localization annotation. *Bachelor Thesis*. Munich: Technical University of Munich; 2017.

[18] The Gene Ontology C (2017). Expansion of the Gene Ontology knowledgebase and resources. Nucleic Acids Res.

[19] Ashburner M, Ball CA, Blake JA, Botstein D, Butler H, Cherry JM, Davis AP, Dolinski K, Dwight SS, Eppig JT (2000). Gene ontology: tool for the unification of biology. The Gene Ontology consortium. Nat Genet.

[20] Andersen JS, Lam YW, Leung AK, Ong SE, Lyon CE, Lamond AI, Mann M (2005). Nucleolar proteome dynamics. Nature.

[21] Comings DE (1972). The structure and function of chromatin. Adv Hum Genet.

[22] Lallemand-Breitenbach V (2010). PML nuclear bodies. Cold Spring Harb Perspect Biol.

[23] Ofran Y, Rost B (2007). Protein-protein interaction hotspots carved into sequences. PLoS Comput Biol.

[24] Heinzinger M (2017). Predicting protein contacts and interactions using co-evolution and deep learning.

[25] Hamp T, Goldberg T, Rost B (2013). Accelerating the original profile kernel. PLoS One.

[26] Keshava Prasad TS, Goel R, Kandasamy K, Keerthikumar S, Kumar S, Mathivanan S, Telikicherla D, Raju R, Shafreen B, Venugopal A (2009). Human protein reference database--2009 update. Nucleic Acids Res.

[27] Mika S, Rost B (2005). NMPdb: database of nuclear matrix proteins. Nucleic Acids Res.

[28] Leung AK, Trinkle-Mulcahy L, Lam YW, Andersen JS, Mann M, Lamond AI (2006). NOPdb: nucleolar proteome database. Nucleic Acids Res.

[29] Dellaire G, Farrall R, Bickmore WA (2003). The nuclear protein database (NPD): sub-nuclear localisation and functional annotation of the nuclear proteome. Nucleic Acids Res.

[30] Willadsen K, Mohamad N, Boden M (2012). NSort/DB: an intranuclear compartment protein database. Genomics Proteomics Bioinformatics.

[31] Bairoch A, Apweiler R (2000). The SWISS-PROT protein sequence database and its supplement TrEMBL in 2000. Nucleic Acids Res.

[32] Mika S, Rost B (2003). UniqueProt: creating representative protein sequence sets. Nucleic Acids Res.

[33] Sander C, Schneider R (1991). Database of homology-derived protein structures and the structural meaning of sequence alignment. Proteins.

[34] Rost B (1999). Twilight zone of protein sequence alignments. Protein Eng.

[35] Olson DL, Delen D. Advanced data mining techniques, 1 edn. eBook: Springer-Verlag Berlin Heidelberg; 2008.

[36] Altschul SF, Madden TL, Schaffer AA, Zhang J, Zhang Z, Miller W, Lipman DJ (1997). Gapped BLAST and PSI-BLAST: a new generation of protein database search programs. Nucleic Acids Res.

[37] Bairoch A, Apweiler R (1997). The SWISS-PROT protein sequence data bank and its supplement TrEMBL. Nucleic Acids Res.

[38] Berman HM, Westbrook J, Feng Z, Gilliland G, Bhat TN, Weissig H, Shindyalov IN, Bourne PE (2000). The Protein Data Bank. Nucleic Acids Res.

[39] Cortes C, Vapnik VN (1995). Support vector networks. Mach Learn.

[40] Chang C-C, Lin C-J (2011). LIBSVM: A library for Support Vector Machines. ACM Trans Intell Syst Technol.

[41] Kuang R, Wang K, Wang K, Siddiqi M, Freund Y, Leslie C. Profile-based string kernels for remote homology detection and motif extraction. Journal of bioinformatics and computational biology. 2005;3(3):527-550. 10.1142/s021972000500120x 16108083

[42] Platt JC. Probabilistic outputs for support vector machines and comparisons to regularized likelihood methods. In: Advances in large margin classifiers: MIT Press; 1999;10(3):61–74.

[43] Gribskov M, Robinson NL (1996). Use of receiver operating characteristic (ROC) analysis to evaluate sequence matching. Comput Chem.

[44] Eden E, Navon R, Steinfeld I, Lipson D, Yakhini Z (2009). GOrilla: a tool for discovery and visualization of enriched GO terms in ranked gene lists. BMC Bioinf.

[45] Benjamini Y, Hochberg Y. Controlling the false discovery rate: a practical and powerful approach to multiple testing. J R Stat Soc Ser B Methodol. 1995;57(1):289–300.

[46] Calderone A, Castagnoli L, Cesareni G (2013). Mentha: a resource for browsing integrated protein-interaction networks. Nat Methods.

[47] Kotlyar M, Pastrello C, Sheahan N, Jurisica I (2016). Integrated interactions database: tissue-specific view of the human and model organism interactomes. Nucleic Acids Res.

[48] Rolland T, Tasan M, Charloteaux B, Pevzner SJ, Zhong Q, Sahni N, Yi S, Lemmens I, Fontanillo C, Mosca R (2014). A proteome-scale map of the human interactome network. Cell.

[49] Das J, Yu H (2012). HINT: high-quality protein interactomes and their applications in understanding human disease. BMC Syst Biol.

[50] Razick S, Magklaras G, Donaldson IM (2008). iRefIndex: a consolidated protein interaction database with provenance. BMC Bioinf.

[51] Li T, Wernersson R, Hansen RB, Horn H, Mercer J, Slodkowicz G, Workman CT, Rigina O, Rapacki K, Staerfeldt HH (2017). A scored human protein-protein interaction network to catalyze genomic interpretation. Nat Methods.

