# Correction to: Detailed prediction of protein sub-nuclear localization

**DOI:** 10.1186/s12859-019-3305-4

**Published:** 2019-12-20

**Authors:** Maria Littmann, Tatyana Goldberg, Sebastian Seitz, Mikael Bodén, Burkhard Rost

**Affiliations:** 1 Department of Informatics, Bioinformatics & Computational Biology - i12, TUM (Technical University of Munich), Boltzmannstr. 3, 85748 Garching/Munich, Germany; 2 School of Chemistry and Molecular Biosciences, UQ (University of Queensland), Cooper Rd, Brisbane City, QLD 4072 Australia; 3 Institute for Advanced Study (TUM-IAS), Lichtenbergstr 2a, 85748 Garching/Munich, Germany; 4 TUM School of Life Sciences Weihenstephan (WZW), Alte Akademie 8, Freising, Germany; 5 Department of Biochemistry and Molecular Biophysics & New York Consortium on Membrane Protein Structure (NYCOMPS), Columbia University, 701 West, 168th Street, New York, NY 10032 USA

**Correction to: BMC Bioinformatics (2019) 20:205**

**https://doi.org/10.1186/s12859-019-2790-9**

Following publication of the original article [1], the author reported that an incorrect figure has been published as Fig. 2. The correct Fig. 2 is shown below.

**Fig. 2:**
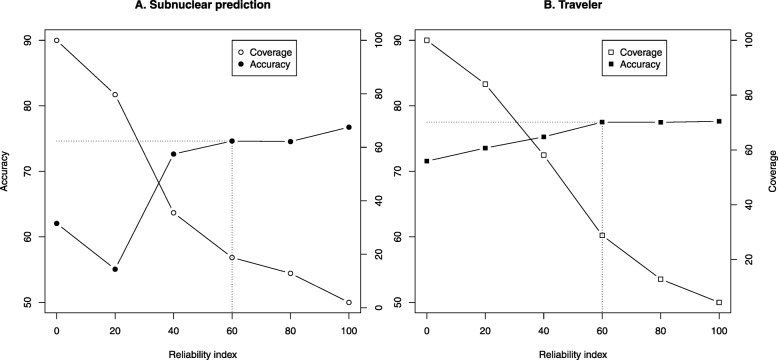
Highly reliable predictions more accurate. The reliability index (RI, x-axis) of *LocNuclei* scaled between 0 (unreliable) and 100 (reliable). It related the prediction strength to the performance. The data for this figure were binned in intervals of 20. Each point reflected the cumulative performance, i.e. we computed accuracy (Q_13_ and Q_2_) and coverage (percentage of proteins for which predictions were made above given RI). **a** For the prediction of 13 nuclear sub-structures, 19% of all proteins were predicted at RI > 60 (point marked by dotted lines). For this top 19%, accuracy rose from the average Q13 = 62% (indicated by leftmost black point) to 75% (point marked by dotted lines). For our data set, RI < 20 did not correlate with accuracy. **b** For the prediction of traveler proteins, 29% of all proteins were predicted at RI > 60 (part B, point marked by dotted lines) with Q2 = 78% (point marked by dotted lines, improving over the average of 72% by six percentage points)

The publisher apologizes to the authors and readers for the inconvenience.
