# Supplementary material for: Detailed prediction of protein sub-nuclear localization
Source: BMC Bioinformatics. 2019 Apr 23;20:205. doi: 10.1186/s12859-019-2790-9 (PMC6480651; doi:10.1186/s12859-019-2790-9)
Supplement: Supplementary file 1 — Figure S1. Effect of E-value thresholds on combined prediction of traveler proteins. Figure S2. Composition of sub-nuclear compartments in the human, chimp, mouse and yeast proteome and LocNuclei’s development set. Table S1. Composition of the sub-nuclear development set for LocNuclei. Table S2. LocNuclei confusion matrix for homology-based inference and machine learning prediction. Table S3. Comparison between LocNuclei and LocNuclei-NSort. Table S4. Euclidean distance between organisms based on subnuclear location spectra predicted with SVM Profile Kernel (de novo) or homology-based inference. Table S5. Top ten statistically enriched GO terms for each sub-nuclear compartment. Table S6. Normalization of sub-nuclear localization terms. Table S7. Chosen hyperparameters for the 14 different SVM Profile Kernels. (DOCX 43170 kb) [file 12859_2019_2790_MOESM1_ESM.docx]

Supporting online material
for:
Detailed prediction of protein sub-nuclear localization

Maria Littmann, Tatyana Goldberg, Sebastian Seitz, Mikael Bodén & Burkhard Rost

# Table of Contents for Supporting Online Material

1. Effect of E-value thresholds on combined prediction of traveler proteins
2. Composition of sub-nuclear compartments for different organisms and LocNuclei’s development set
3. Normalization of sub-nuclear localization terms
4. Composition of the sub-nuclear development set
5. Comparison between LocNuclei and LocNuclei-NSort
6. Euclidean distance between organisms based on subnuclear location spectra
7. Top ten statistically enriched GO terms

# Short description of Supporting Online Material

1. Figure showing Q2 for different E-value thresholds for the homology-based component of LocNuclei for traveler predictions
2. Figure showing the composition of sub-nuclear compartments (without correction for prediction bias) for different organisms and LocNuclei’s development set
3. Table showing the normalization of sub-nuclear terms
4. Table showing the composition of the sub-nuclear development set for LocNuclei across 13 sub-nuclear classes
5. Table showing AUC values for LocNuclei and LocNuclei-NSort
6. Table showing the Euclidean distance between organisms based on predicted subnuclear location spectra
7. Table showing the top ten statistically enriched GO terms for every sub-nuclear compartment from the GO enrichment analysis

# Material

**Fig. S1:**





**Fig. S1: Effect of E-value thresholds on combined prediction of traveler proteins.** The accuracy $Q_{2}$ for classifying nuclear proteins as travelers or not using homology-based inference with PSI-BLAST (based on 12,055 experimentally annotated nuclear proteins) varies at different E-value thresholds (darker grey bars on the left). For proteins for which a protein with experimentally known nuclear sub-structure annotation is more sequence similar than the threshold, performance depends on the threshold (black line). Homology based inference reaches the highest accuracy of Q2 $=77\%$ at the stringent E-value $\leq{10}^{-50}$.However, when evaluated on the entire test set (*i.e.* also on proteins for which no homolog is available), the performance drops significantly to Q2 = 53% compared to a random prediction of Q2 = 49%. The performance of the SVM on the same set, however, reaches Q2 = 66% (the performance is marked by grey lines). The lighter grey bars mark the combination of homology inference and machine learning. The optimal threshold for the combination was E-value ≤ ${10}^{-5}$. One standard error marked on each bar and on the black line and through the dotted lines for ML.

### Fig. S2:


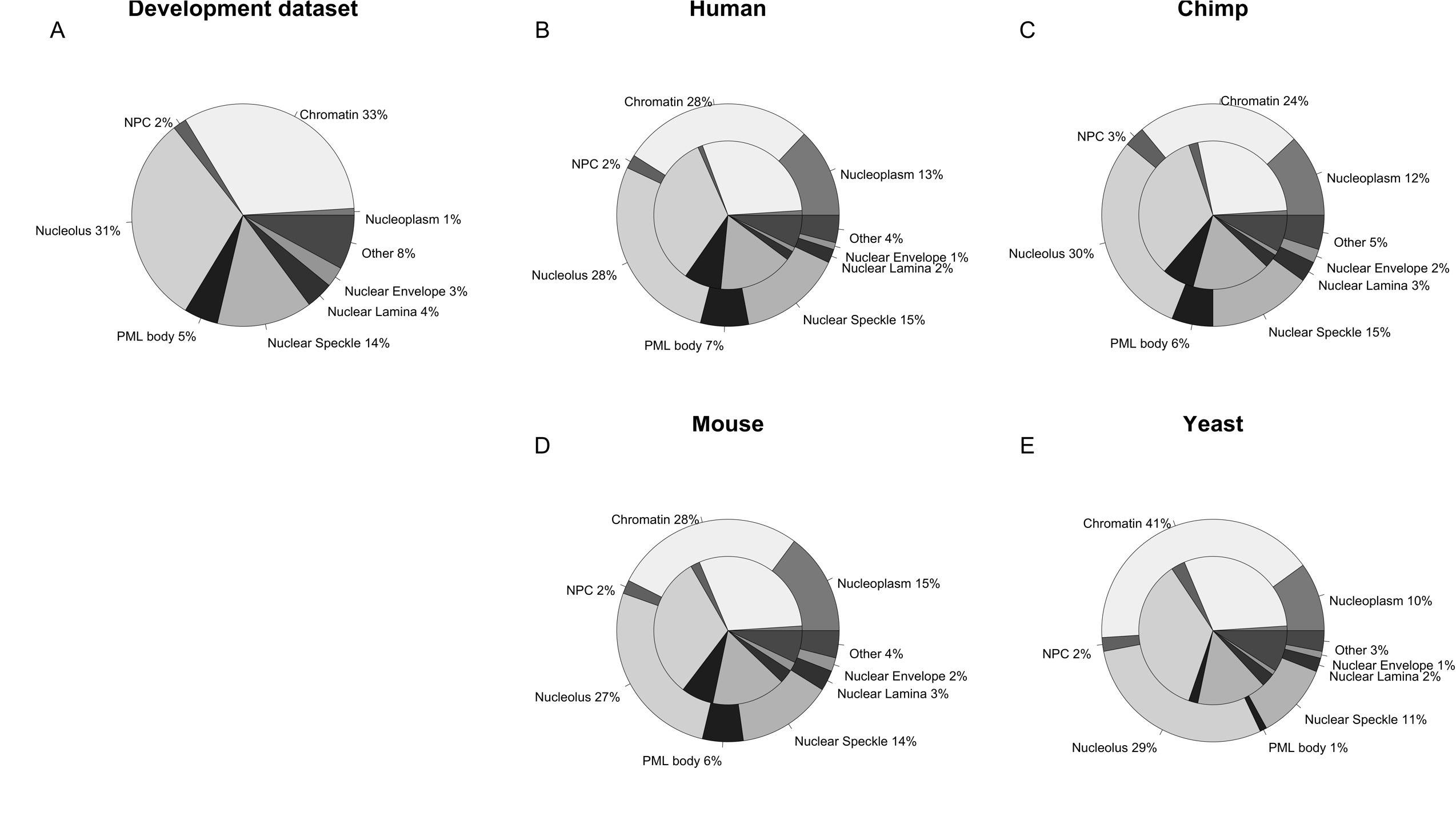


**Fig. S2:** **Composition of sub-nuclear compartments in the human, chimp, mouse and yeast proteome and LocNuclei’s development set.** A. Composition of LocNuclei’s development set (assembled from nuclear proteins of various organisms) B. Composition of 5,088 human nuclear C. Composition of 4,067 nuclear proteins from chimp. D. Composition of 6,041 nuclear proteins from mouse E. Composition of 1,790 nuclear proteins from yeast. All nuclear proteome sets were obtained by taking nuclear and nuclear membrane proteins from LocTree3 [29] whole proteome prediction. Differences in the number of nuclear proteins identified from LocTree3 and numbers given here originate from the fact that LocNuclei was not able to predict any sub-nuclear compartment for some of the proteins. For each organism, the outer circle shows the composition of the whole dataset while the inner circle only shows the composition of the proteins predicted using homology inference. Human, mouse and chimp are very similar because they also share a large amount of homologous proteins while yeast with a more different proteome in general also shows a different composition of the nuclear proteome.

Table S1: Composition of the sub-nuclear development set for LocNuclei

|  | *Chromatin (697)* | *Nucleolus (653)* | *Nuclear speckle (292)* | *PML body (95)* | *Nuclear lamina (80)* | *Nuclear matrix (74)* | *Nuclear envelope (72)* | *Cajal body (42)* | *Nuclear pore complex (35)* | *Nucleoplasm (29)* | *Kinetochore (25)* | *Spindle apparatus (14)* | *Perinucleolar (13)* |
| --- | --- | --- | --- | --- | --- | --- | --- | --- | --- | --- | --- | --- | --- |
| *Chromatin (697)* | **584** |  |  |  |  |  |  |  |  |  |  |  |  |
| *Nucleolus (653)* | 68 | **483** |  |  |  |  |  |  |  |  |  |  |  |
| *Nuclear speckle (292)* | 22 | 79 | **176** |  |  |  |  |  |  |  |  |  |  |
| *PML body (95)* | 23 | 18 | 9 | **49** |  |  |  |  |  |  |  |  |  |
| *Nuclear lamina (80)* | 5 | 8 | 2 | 3 | **51** |  |  |  |  |  |  |  |  |
| *Nuclear matrix (74)* | 4 | 3 | 3 | 2 | 1 | **63** |  |  |  |  |  |  |  |
| *Nuclear envelope (72)* | 2 | 0 | 0 | 1 | 3 | 1 | **63** |  |  |  |  |  |  |
| *Cajal body (42)* | 3 | 15 | 14 | 4 | 2 | 0 | 0 | **15** |  |  |  |  |  |
| *Nuclear pore complex (35)* | 5 | 6 | 3 | 2 | 17 | 0 | 0 | 2 | **12** |  |  |  |  |
| *Nucleoplasm (29)* | 3 | 8 | 3 | 1 | 0 | 2 | 2 | 0 | 0 | **13** |  |  |  |
| *Kinetochore (25)* | 3 | 4 | 0 | 0 | 1 | 0 | 0 | 0 | 2 | 1 | **15** |  |  |
| *Spindle apparatus (14)* | 2 | 1 | 0 | 1 | 4 | 1 | 1 | 0 | 1 | 0 | 3 | **6** |  |
| *Perinucleolar (13)* | 3 | 4 | 6 | 2 | 1 | 0 | 0 | 1 | 0 | 0 | 3 | 0 | **2** |

The table displays numbers of sequence-unique proteins (HVAL [7, 8] $\leq20$) across 13 sub-nuclear localization classes in the development set of LocNuclei. Only proteins with experimental annotations extracted from HPRD [1], NMPdb [2], NOPdb [3], NPD [4], NSort/DB [5] and Swiss-Prot [6] are used. The numbers of unique sequences per localization are given in parentheses. The numbers on the diagonal describe sequences with the annotation of one localization class (e.g. 584 sequences in the set were annotated to localize at the chromatin only). Other numbers are annotations of two sub-nuclear compartments. Note that some sequences had annotations of more than two compartments.

Table S2: LocNuclei confusion matrix for homology-based inference and machine learning prediction

| Observed:->  Predicted: | *Chromatin* | *Nucleolus* | *Nuclear speckle* | *PML body* | *Nuclear lamina* | *Nuclear matrix* | *Nuclear envelope* | *Cajal body* | *Nuclear pore complex* | *Nucleoplasm* | *Kinetochore* | *Spindle apparatus* | *Perinucleolar* | *SUM predicted* |
| --- | --- | --- | --- | --- | --- | --- | --- | --- | --- | --- | --- | --- | --- | --- |
| *Chromatin* | **208**  **298** | 19  13 | 4  7 | 4  3 | 0  0 | 3  3 | 1  2 | 0  1 | 0  1 | 3  1 | 4  2 | 0  1 | 1  0 | 247  332 |
| *Nucleolus* | 28  21 | **212**  **249** | 17  12 | 8  3 | 0  4 | 5  7 | 0  3 | 2  4 | 1  1 | 2  1 | 1  1 | 0  1 | 1  1 | 277  308 |
| *Nuclear speckle* | 4  10 | 11  8 | **66**  **87** | 1  1 | 0  2 | 3  1 | 0  1 | 0  1 | 0  0 | 1  1 | 1  0 | 0  0 | 0  0 | 87  112 |
| *PML body* | 1  11 | 2  11 | 3  6 | **21**  **17** | 0  2 | 0  3 | 0  2 | 0  1 | 1  0 | 0  0 | 1  0 | 0  0 | 0  0 | 29  53 |
| *Nuclear lamina* | 1  4 | 1  5 | 2  1 | 1  1 | **12**  **29** | 2  1 | 5  2 | 0  0 | 1  1 | 0  0 | 1  0 | 0  0 | 0  0 | 26  44 |
| *Nuclear matrix* | 3  4 | 6  3 | 4  1 | 3  1 | 1  1 | **16**  **9** | 0  1 | 0  0 | 0  0 | 0  1 | 0  0 | 0  0 | 0  0 | 33  21 |
| *Nuclear envelope* | 1  3 | 0  4 | 0  1 | 0  0 | 2  1 | 0  1 | **14**  **20** | 0  0 | 5  1 | 0  0 | 1  0 | 0  0 | 0  0 | 23  31 |
| *Cajal body* | 0  3 | 2  3 | 0  2 | 0  0 | 0  1 | 0  1 | 0  0 | **8**  **2** | 0  0 | 1  0 | 0  0 | 0  0 | 0  0 | 11  12 |
| *Nuclear pore complex* | 0  4 | 1  3 | 0  1 | 1  1 | 2  1 | 0  0 | 5  1 | 0  0 | **10**  **5** | 0  0 | 1  0 | 0  0 | 0  0 | 20  16 |
| *Nucleoplasm* | 1  38 | 3  35 | 2  28 | 2  9 | 0  7 | 0  8 | 0  5 | 0  10 | 0  3 | **5**  **8** | 0  2 | 0  1 | 0  2 | 13  156 |
| *Kinetochore* | 2  2 | 3  2 | 0  0 | 1  0 | 0  1 | 0  0 | 0  1 | 0  1 | 2  0 | 1  1 | **3**  **2** | 0  0 | 0  0 | 12  10 |
| *Spindle apparatus* | 0  32 | 1  26 | 0  19 | 0  8 | 0  10 | 0  9 | 0  7 | 0  5 | 0  1 | 0  1 | 0  1 | **1**  **7** | 0  1 | 2  127 |
| *Perinucleolar* | 0  0 | 2  1 | 0  0 | 0  0 | 0  0 | 0  0 | 0  0 | 0  1 | 0  0 | 0  0 | 0  0 | 0  0 | **4**  **0** | 6  2 |
| *None* | 18 | 27 | 29 | 9 | 4 | 2 | 2 | 6 | 1 | 2 | 4 | 3 | 3 | 110 |
| *% observed* | *33* | *31* | *14* | *4* | *4* | *3* | *3* | *2* | *2* | *1* | *1* | *1* | *1* |  |
| ***SUM observed*** | **697** | **653** | **292** | **95** | **80** | **74** | **72** | **42** | **34** | **29** | **25** | **14** | **13** |  |

The confusion matrix for LocNuclei predictions on the development set with the columns showing the number of observed and the rows the number of predicted proteins. In each cell, the upper numbers are always predictions through homology-based inference and lower numbers are predictions with the SVM Profile Kernel.

Table S3: Comparison between LocNuclei and LocNuclei-NSort

| *Sub-nuclear compartment* | *Number of proteins* | *AUC LocNuclei-NSort* | *AUC LocNuclei* |
| --- | --- | --- | --- |
| Perinucleolar | 13 | 0.71$\pm$0.04 | 0.83$\pm$0.03 |
| Cajal body | 35 | 0.64$\pm$0.02 | 0.55$\pm$0.03 |
| Nuclear pore complex | 30 | 0.85$\pm$0.02 | 0.83$\pm$0.02 |
| Nuclear lamina | 41 | 0.78$\pm$0.02 | 0.76$\pm$0.02 |
| PML bodies | 68 | 0.74$\pm$0.01 | 0.73$\pm$0.02 |
| Chromatin | 204 | 0.75$\pm$0.01 | 0.73$\pm$0.01 |
| Nuclear speckle | 250 | 0.76$\pm$0.01 | 0.74$\pm$0.01 |
| Nucleolus | 409 | 0.72$\pm$0.01 | 0.70$\pm$0.01 |
| Sum/Mean | 849 | 0.74$\boldsymbol{\pm}$0.02 | 0.73$\boldsymbol{\pm}$0.02 |

Comparing the original version of *LocNuclei* trained on 13 compartments and *LocNuclei-NSort* trained on the development set of NSort and predicting 8 compartments shows that LocNuclei can perform equally well. It is even better on proteins in the perinucleolar than LocNuclei-NSort while being worse on proteins located in the cajal bodies.

Table S4: Euclidean distance between organisms based on subnuclear location spectra predicted with SVM Profile Kernel (de novo) or homology-based inference

|  | De novo prediction | | | | Homology-based inference | | | |
| --- | --- | --- | --- | --- | --- | --- | --- | --- |
|  | Human | Chimp | Mouse | Yeast | Human | Chimp | Mouse | Yeast |
| Human | 0 | 7.2 | 1.9 | 12.8 | 0 | 1.7 | 2.8 | 3.9 |
| Chimp | 7.2 | 0 | 6.1 | 18.7 | 1.7 | 0 | 2.7 | 4.0 |
| Mouse | 1.9 | 6.1 | 0 | 13.3 | 2.8 | 2.7 | 0 | 4.6 |
| Yeast | 12.8 | 18.7 | 13.3 | 0 | 3.9 | 4.0 | 4.6 | 0 |

We calculate the Euclidean distance between predicted subnuclear location spectra and use that distance as proxy to identify evolutionary relationships. Comparing the results for location spectra predicted with an SVM Profile Kernel (*de novo*) to those predicted with homology-based inference shows that *de novo* prediction cannot entirely capture the expected relationships, i.e. the distance between human and mouse is smaller than between human and chimp. Location spectra predicted with homology-based inference succeed in reflecting the expected evolutionary relationship, i.e. the distance between human and chimp is the smallest

Table S5: Top ten statistically enriched GO terms for each sub-nuclear compartment

| *Compart-ment* | *Top 10 enriched*  *GO terms* | *Description* | *No. of enriched GO terms* | *No. of proteins in this compart-ment* |
| --- | --- | --- | --- | --- |
| *Nuclear envelope* | GO:0006998  GO:0061024  GO:0007077  GO:0030397  GO:0051081  GO:0006409  GO:0051031  GO:0097064  GO:0075733  GO:0046794 | Nuclear envelope organization  Membrane organization  Mitotic nuclear envelope disassembly  Membrane disassembly  Nuclear envelope disassembly  tRNA export from nucleus  tRNA transport  ncRNA export from nucleus  Intracellular transport of virus  Transport of virus | 99 | 58 |
| *Chromatin* | GO:0006325  GO:2001141  GO:1903506  GO:0006355  GO:0034645  GO:0097659  GO:0006351  GO:0031326  GO:0009889  GO:0010556 | Chromatin organization  Regulation of RNA biosynthetic process  Regulation of nucleic acid-templated transcription  Regulation of transcription, DNA-templated  Cellular macromolecule biosynthetic process  Nucleic acid-templated transcription  Transcription, DNA-templated  Regulation of cellular biosynthetic process  Regulation of biosynthetic process  Regulation of macromolecule biosynthetic process | 97 | 1901 |
| *Nuclear pore complex* | GO:0006606  GO:0017038  GO:0006913  GO:0051169  GO:0051170  GO:0051168  GO:0006409  GO:0051031  GO:0034504  GO:0071705 | Protein import into nucleus  Protein import  Nucleocytoplasmic transport  Nuclear transport  Import into nucleus  Nuclear export  tRNA export from nucleus  tRNA transport  Protein localization to nucleus  Nitrogen compound transport | 77 | 141 |
| *Nuclear speckle* | GO:0008380  GO:0006397  GO:0016071  GO:0000375  GO:0000377  GO:0000398  GO:0006396  GO:0043484  GO:0048024  GO:0050684 | RNA splicing  mRNA processing  mRNA metabolic process  RNA splicing via transesterification reactions  RNA splicing via transesterification reactions with bulged adenosine  mRNA splicing via spliceosome  RNA processing  Regulation of RNA splicing  Regulation of mRNA splicing via spliceosome  Regulation of mRNA processing | 71 | 976 |
| *PML body* | GO:0043401  GO:0009755  GO:0070936  GO:0006357  GO:0006355  GO:0045944  GO:1903506  GO:2001141  GO:0030522  GO:0048522 | Steroid hormone mediated signalling pathway  Hormone-mediated signalling pathway  Protein K48-linked ubiquitination  Regulation of transcription by RNA polymerase II  Regulation of transcription, DNA-templated  Positive regulation of transcription by RNA polymerase II  Regulation of nucleic acid-templated transcription  Regulation of RNA biosynthetic process  Intracellular receptor signalling pathway  Positive regulation of cellular process | 64 | 470 |
| *Cajal body* | GO:0060333  GO:0060337  GO:0016074  GO:0043170  GO:0006807  GO:0044238  GO:0071704  GO:0044237  GO:0000387  GO:0031118 | Interferon-gamma-mediated signalling pathway  Type I interferon signalling pathway  snoRNA metabolic process  Macromolecule metabolic process  Nitrogen compound metabolic process  Primary metabolic process  Organic substance metabolic process  Cellular metabolic process  Spliceosomal snRNP assembly  rRNA pseudouridine synthesis | 62 | 67 |
| *Nucleolus* | GO:0006364  GO:0016072  GO:0034470  GO:0034660  GO:0006396  GO:0044085  GO:0022613  GO:0009451  GO:0030490  GO:0006399 | rRNA processing  rRNA metabolic process  ncRNA processing  ncRNA metabolic process  RNA processing  Cellular component biogenesis  Ribonucleoprotein complex biogenesis  RNA modification  Maturation of SSU-rRNA  tRNA metabolic process | 54 | 1856 |
| *Kine-tochore* | GO:0007062  GO:0051301  GO:0007059  GO:0051276  GO:0000819  GO:0008608  GO:0000070  GO:0098813  GO:0071173  GO:0071174 | Sister chromatid cohesion  Cell division  Chromosome segregation  Chromosome organization  Sister chromatid segregation  Attachment of spindle microtubules to kinetochore  Mitotic sister chromatid segregation  Nuclear chromosome segregation  Spindle assembly checkpoint  Mitotic spindle checkpoint | 38 | 42 |
| *Nuclear matrix* | GO:0021515  GO:0009725  GO:0009719  GO:0097485  GO:0007411  GO:0021527  GO:0045944  GO:1904903  GO:1904896  GO:0045935 | Cell differentiation in spinal cord  Response to hormone  Response to endogenous stimulus  Neuron projection guidance  Axon guidance  Spinal cord association neuron differentiation  Positive regulation of transcription by RNA polymerase II  ESCRT III complex disassembly  ESCRT complex disassembly  Positive regulation of nucleobase-containing compound metabolic process | 38 | 120 |
| *Nuclear lamina* | GO:0006998  GO:0007010  GO:0061024  GO:0051225  GO:0007030  GO:0007051  GO:0000226  GO:0090286  GO:0007017  GO:0051179 | Nuclear envelope organization  Cytoskeleton organization  Membrane organization  Spindle assembly  Golgi organization  Spindle organization  Microtubule cytoskeleton organization  Cytoskeletal anchoring at nuclear membrane  Microtubule-based process  Localization | 25 | 130 |
| *Peri-nucleolar* | GO:0048010  GO:0045445 | Vascular endothelial growth factor receptor signalling pathway  Myoblast differentiation | 2 | 33 |
| *Nucleo-plasm* | GO:0031424 | Keratinization | 1 | 852 |
| *Spindle apparatus* |  |  | 0 (lowest p-value= 0.0247) | 13 |
| *Traveler* | GO:0051179  GO:0051234  GO:0006810  GO:0008104  GO:0015833  GO:0042886  GO:0015031 GO:0033036  GO:0045184  GO:0016192 | Localization  Establishment of localization  Transport  Protein localization  Peptide transport  Amide transport  Protein transport  Macromolecule localization  Establishment of protein localization  Vesicle-mediated transport | 207 | 2248 |

The table displays the overall number of enriched GO terms and the top ten enriched GO terms for each sub-nuclear compartment and for proteins predicted as traveler. GO enrichment analysis was performed for the 5,088 proteins of the human nuclear proteome and their sub-nuclear localizations as predicted by LocNuclei. A GO term is considered as enriched if the p-value, adjusted for multiple testing, is < 0.01. Terms are ranked by p-value where the first term is the term with the lowest p-value. For Spindle apparatus, there are no enriched terms which is probably due to the low number of proteins prediction for this localization (13). For compartments with many proteins and a clearly defined function like nucleolus, chromatin, kinetochores, nuclear envelope or nuclear pore complex, the enriched GO terms reflect the expected functionality. Also for traveler proteins, the enriched GO terms all associated with transport and localization give evidence about the functionality of these proteins.

Table S6: Normalization of sub-nuclear localization terms

| Databases term | Normalized term |
| --- | --- |
| Cajal body, cajal bodies, gem | Cajal bodies |
| Chromatin, centromere, chromosome, heterochromatin, telomere, unsynapsed chromosome axes | Chromatin |
| Nuclear envelope, nuclear membrane, nucleus membrane | Nuclear envelope |
| Nuclear lamina, nuclear periphery, nucleus lamina | Nuclear lamina |
| Nuclear matrix, nucleus matrix | Nuclear matrix |
| Nuclear pore | Nuclear pore complex |
| Nuclear speckle | Nuclear speckles |
| Nucleolus, nucleolar | Nucleolus |
| Nucleoplasm | Nucleoplasm |
| Perinucleolar | Perinucleolar compartment |
| PML body, nuclear dots, PML-NBs, PML/ND10 bodies | PML bodies |
| Kinetochore | Kinetochore |
| Spindle apparatus, spindle microtubules, spindle midzone, spindle poles | Spindel apparatus |

Databases HPRD [1], NMPdb [2], NOPdb [3], NPD [4], NSort/DB [5] and Swiss-Prot [6] annotate sub-nuclear proteins using synonyms for some terms. We extracted these terms and normalized them to 13 sub-nuclear localization classes. The normalization was done case-insensitive; terms of the same class are separated by comma.

Table S7: Chosen hyperparameters for the 14 different SVM Profile Kernels

|  | k | σ | C | tol |
| --- | --- | --- | --- | --- |
| Cajal Body | 4 | 7 | 1.0 | 0.0001 |
| Chromatin | 4 | 7 | 2.0 | 0.0001 |
| Kinetochore | 5 | 8 | 1.0 | 0.0001 |
| Nuclear Envelope | 4 | 7 | 2.0 | 0.1 |
| Nuclear Lamina | 4 | 7 | 2.0 | 0.1 |
| NPC | 3 | 5 | 2.0 | 0.1 |
| Nuclear speckle | 3 | 5 | 2.0 | 0.1 |
| Nuclear matrix | 4 | 6 | 1.0 | 0.0001 |
| Nucleolus | 4 | 9 | 2.0 | 0.0001 |
| Nucleoplasm | 3 | 7 | 0.5 | 0.0001 |
| PML body | 4 | 8 | 2.0 | 0.0001 |
| Perinucleolar | 4 | 7 | 2.0 | 0.0001 |
| Spindle apparatus | 4 | 7 | 1.0 | 0.0001 |
| Traveler | 3 | 6 | 0.1 | 0.1 |

For the Profile Kernel, we optimized the parameters *k*, the k-mer length, and *γ*, the conservation threshold. For the SVMs, we focused on optimizing *C*, the penalty parameter of the error term, and *tol*, the tolerance for the stopping criterion. We optimized all parameters for the 14 SVMs independently.

# References for Supporting Online Material

1. Keshava Prasad, T.S., et al., *Human Protein Reference Database--2009 update.* Nucleic Acids Res, 2009. **37**(Database issue): p. D767-72.

2. Mika, S. and B. Rost, *NMPdb: Database of Nuclear Matrix Proteins.* Nucleic Acids Res, 2005. **33**(Database issue): p. D160-3.

3. Leung, A.K., et al., *NOPdb: Nucleolar Proteome Database.* Nucleic Acids Res, 2006. **34**(Database issue): p. D218-20.

4. Dellaire, G., R. Farrall, and W.A. Bickmore, *The Nuclear Protein Database (NPD): sub-nuclear localisation and functional annotation of the nuclear proteome.* Nucleic Acids Res, 2003. **31**(1): p. 328-30.

5. Willadsen, K., N. Mohamad, and M. Boden, *NSort/DB: an intranuclear compartment protein database.* Genomics Proteomics Bioinformatics, 2012. **10**(4): p. 226-9.

6. Bairoch, A. and R. Apweiler, *The SWISS-PROT protein sequence database and its supplement TrEMBL in 2000.* Nucleic Acids Res, 2000. **28**(1): p. 45-8.

7. Sander, C. and R. Schneider, *Database of homology-derived protein structures and the structural meaning of sequence alignment.* Proteins, 1991. **9**(1): p. 56-68.

8. Rost, B., *Twilight zone of protein sequence alignments.* Protein Eng, 1999. **12**(2): p. 85-94.
